# Long-term oncologic outcomes following breast cancer surgery in adolescents and young adults: a single-center retrospective analysis

**DOI:** 10.3389/fonc.2024.1364608

**Published:** 2024-06-24

**Authors:** Xin Liu, Zengyan Ma, Hongwu Chu, Weihong Nie, Guoxin Sun, Kaihua Zhao, Xiao Zou

**Affiliations:** ^1^ Qingdao Medical College, Qingdao University, Qingdao, China; ^2^ Department of Pathology, Qingdao Central Hospital, Qingdao, China; ^3^ Guangdong Provincial Key Laboratory of Digestive Cancer Research, The Seventh Affiliated Hospital of Sun Yat-sen University, Shenzhen, China; ^4^ Department of Breast Surgery, Qingdao Central Hospital, Qingdao, China; ^5^ Department of Breast Surgery, Xiangdong Hospital Affiliated to Hunan Normal University, Liling, China

**Keywords:** breast cancer, age, young, clinicopathological characteristics, prognosis

## Abstract

**Background:**

Breast cancer (BC) in adolescents and young adults (AYAs, aged 15–39 years), remains inadequately understood. The incidence of BC in AYAs has been steadily increasing, making it the second leading cause of cancer-related mortality among females aged 0–39 globally. This study aimed to elucidate the clinical characteristics and long-term outcomes of AYAs and older adults (OAs, aged > 39 years) with BC who underwent surgery.

**Methods:**

From January 2011 to June 2017, BC patients who underwent surgery were enrolled in this study and divided into AYA group and OA group. Clinical characteristics, recurrence-free survival (RFS), and overall survival (OS) were compared between these two groups, both before and after propensity score matching (PSM). Univariate and multivariate Cox proportional hazard regression analyses were performed to assess the influence of age on OS and RFS.

**Results:**

Compared to the OA group, the AYA group exhibited a younger age at menarche (p < 0.001), a lower prevalence of menopausal status (p < 0.001), a reduced occurrence of comorbid conditions (p < 0.001), fewer instances of undergoing mastectomy (p = 0.031), a higher incidence of Triple-Negative Breast Cancer (TNBC) (p = 0.046), and elevated Ki-67 levels (p = 0.036). In terms of prognostic outcomes, within the study cohort, AYAs had a higher mortality rate and poorer long-term survival compared to OAs, both before and after PSM. In the PSM cohort, AYAs experienced a significantly shorter mean OS (p < 0.001) and RFS (p < 0.001). Young age (15–39 years) emerged as an independent risk factor for OS (HR 2.659, 95% CI 1.385–5.106, p = 0.003) and RFS (HR 3.235, 95% CI 2.085–5.022, p < 0.001) in BC patients following surgery.

**Conclusion:**

Significant differences were identified in the clinicopathological characteristics between AYA and OA patients with BC. In comparison to OA patients, AYA patients exhibited a less favorable long-term prognosis, with young age emerging as an independent prognostic risk factor for both OS and RFS in BC patients following surgery. Further investigations are warranted to develop age-specific therapeutic approaches for AYA BC patients.

## 1 Introduction

Breast cancer (BC) has surpassed lung cancer in incidence, becoming the most common cancer among women and a significant contributor to cancer-related deaths in the female population. Globally, one-fourth of cancer cases and one-sixth of female cancer-related deaths are attributed to BC (1). While BC is more common in women aged 50 or older, the incidence of BC in young women is on the rise. Compared to older women, young women face a greater risk of developing more aggressive and advanced breast cancer. Currently, it ranks as the second leading cause of cancer-related deaths among women aged 0–39 worldwide, leading to 44,800 annual fatalities (2–4). Studies have indicated that breast cancer in young women exhibits unique, more aggressive, and complex biological characteristics. Consequently, a diagnosis of breast cancer at a young age is associated with unfavorable clinicopathologic attributes and leads to poorer outcomes when compared to older women (5–9). Young age at the diagnosis of breast cancer has been identified globally as an independent factor associated with a higher risk of relapse and mortality in several large studies, even when more aggressive treatments are applied (10). Nonetheless, BC in younger women remains inadequately understood to this day, and management strategies and options are not age specific. Undoubtedly, there is a pressing need to develop a tailored treatment approach for younger women with BC (11). As per the National Cancer Institute of the United States, individuals diagnosed between the ages of 15 and 39 are defined as adolescents and young adults (AYAs) (12). AYAs represent a distinct population separate from children and middle-aged to elderly individuals (13). In terms of both internal and external risk factors contributing to cancer development, tumor biology and prognostic outcomes, cancers that arise in AYAs exhibit distinctions from other age categories (14). Nonetheless, there is a dearth of age-specific clinical features or outcome data for AYAs, and minimal information regarding clinicopathological characteristics and prognosis outcomes in AYAs with BC. The objective of this study was to compare and analyze the clinicopathological characteristics and prognosis outcomes of BC patients in the AYAs and older adults (OAs) groups, with a specific focus on assessing the influence of age at disease onset on BC prognosis.

## 2 Materials and methods

### 2.1 Study population

**Figure 1:**
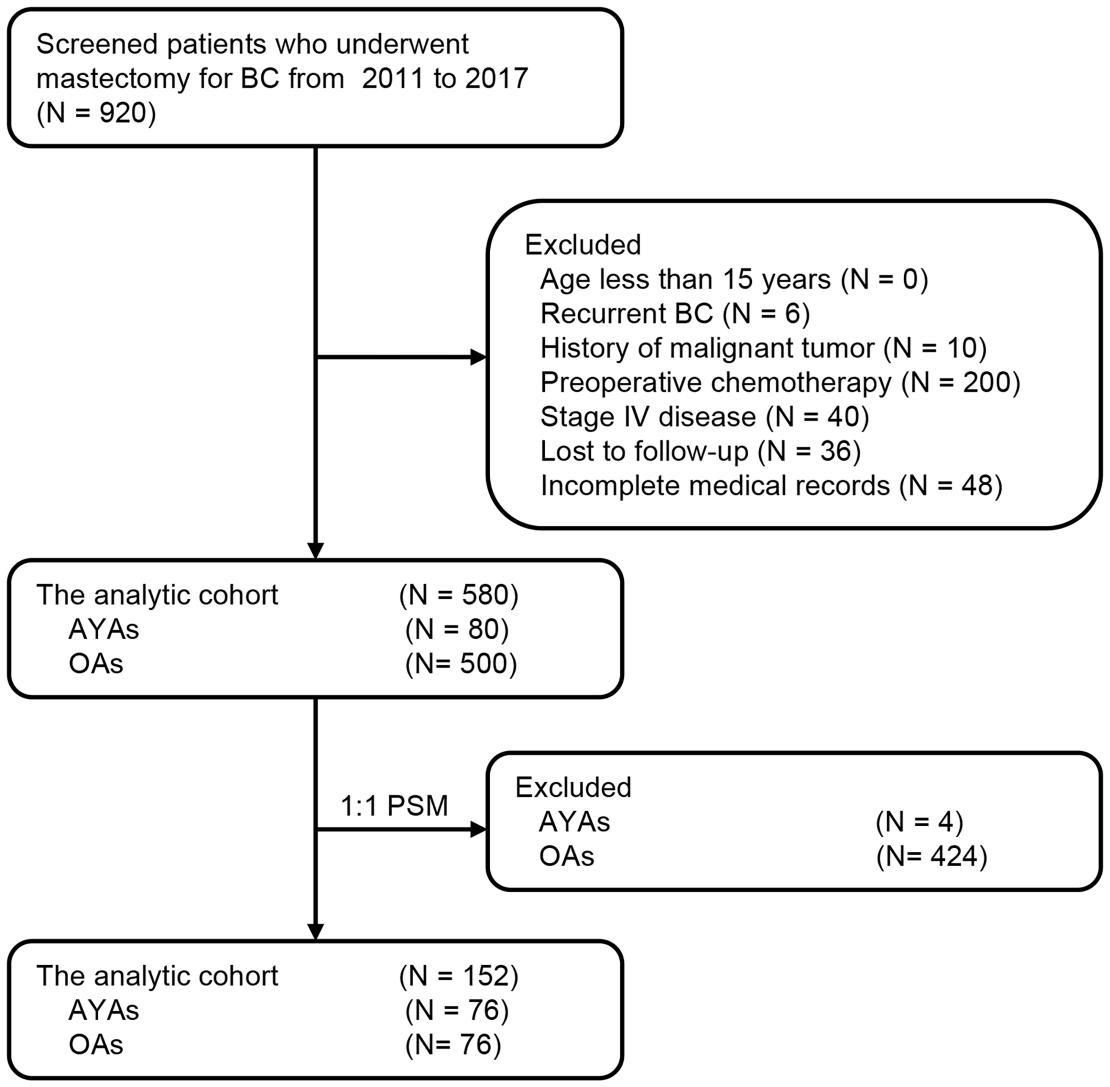
Selection of the study population.

Approved by the Institutional Review Committee of Qingdao Central Hospital, patients diagnosed with BC and undergoing surgery from January 2011 to June 2017 were selected. Among them, 580 patients provided follow-up data and were included for further analysis. Patients were stratified by age, and a retrospective analysis of the study’s patient data was performed. **Figure 1** depicts the patient flowchart. Individuals were divided into two age groups: AYAs (aged 15–39 years, n = 80; 13.8%) and OAs (aged > 39 years, n = 500; 86.2%). The age of disease onset was determined as the age at the initiation of treatment, and the diagnosis of BC was verified through postoperative pathological examination. Exclusion criteria encompassed individuals under the age of 15, those with a prior history of recurrent BC, individuals with a history of malignancies, patients who underwent preoperative neoadjuvant chemotherapy, and individuals who were lost to follow-up. Furthermore, patients with incomplete or missing clinical case data were also excluded. Clinical-pathological and prognostic factors were compared between the two groups. In the survival analysis, patients in stage IV or with unknown disease stages were omitted from the study. This research adhered to the principles of the Helsinki Declaration, and all participating patients provided written informed consent.

### 2.2 Clinicopathological characteristics

Baseline characteristics among patients in the AYA and OA cohort encompassed age, menarche, menopause, abortion, comorbid conditions, Body Mass Index (BMI), tumor location, surgery, tumor size, T stage, N stage, TNM stage, lymphatic invasion, cancer embolus, histological type, histological grade, molecular subtype, hormone receptor status, human epidermal growth factor receptor 2 (HER2) status, Ki-67, and adjuvant therapy. The TNM stage was determined following the guidelines of the eighth edition of the American Joint Committee on Cancer staging system (15). The histological grade was assessed following the Classification of Tumors of the Breast and Female Genital Organs (16). HER2-positivity is defined as a positive outcome in immunohistochemical staining at a 3+ level or in fluorescence *in situ* hybridization (FISH) testing. Hormone receptor (estrogen receptor [ER]/progesterone receptor [PR]) positivity is characterized by ≥ 1% of tumor cell nuclei showing a positive result in immunohistochemical analysis. The luminal subtype is defined as being ER and/or PR positive but HER2 negative. The Triple-Negative Breast Cancer (TNBC) subtype is defined as being negative for ER, PR, and HER2.

### 2.3 Postoperative follow-up

Patients included in the study underwent routine follow-up assessments. In the initial 2 years, patients underwent follow-up assessments every 3 months, followed by semi-annual evaluations for the subsequent 3 years, and subsequently, annual assessments. Postoperative monitoring for tumor recurrence included physical examination, serum tumor marker evaluation, abdominal pelvic ultrasound, breast ultrasound, mammography, and chest CT scan. In addition, patients with high-risk factors such as more than 4 positive lymph nodes underwent whole-body bone scan examination. Patients receiving tamoxifen were also scheduled for annual pelvic examination. The dates of the initial recurrence, last follow-up, and mortality were documented.

### 2.4 Study endpoints and propensity score matching analyses

The endpoints of the study were overall survival (OS) and recurrence-free survival (RFS) of patients. The OS was calculated from the date of surgery to the time of death due to any cause or the last follow-up, while the RFS was calculated from the date of surgery to the time of breast cancer recurrence or the last follow-up. We employed the PSM method as described by Rubin and Rosenbaum to match the AYA and OA patients (17, 18), with the matching process performed using R language software version 4.2.2. The propensity score for everyone was computed through a logistic regression model that considered factors including menarche, menopause, comorbid conditions, BMI, tumor location, surgery, T stage, N stage, TNM stage, tumor size, lymphatic invasion, cancer embolus, histological type, histological grade, molecular subtype, and adjuvant therapy. To minimize conditional bias, we employed a 1:1 non-replacement nearest neighbor matching method based on a greedy algorithm, pairing each patient in the AYA group with the closest OA patient in terms of propensity scores. Various caliper widths and standardized mean differences were utilized to assess the matching process and evaluate the balance of covariate distribution between the two groups. Ultimately, a caliper width of 0.1 was chosen to fulfill the criteria for enhancing homogeneity and minimizing sample loss.

### 2.5 Statistical analyses

Statistical analyses were conducted using IBM SPSS Statistics 26.0 software (SPSS, Inc., Chicago, IL, USA). Categorical variables were presented as counts (percentages), and continuous variables were reported as either mean ± standard deviation (SD). We used appropriate statistical tests to compare continuous variables, including Student’s t-test or Mann-Whitney U test. For categorical variables, we employed Pearson’s chi-squared test or Fisher’s exact test. Survival curves were constructed using the Kaplan-Meier method, and the log-rank test was used to compare OS and RFS between the AYA and OA groups. Univariate and multivariate Cox proportional hazards regression analyses was conducted to estimate hazard ratios and 95% confidence intervals for OS and RFS while identifying independent predictors in BC patients. Variables with a p-value below 0.1 in the univariate analysis were considered for inclusion in the multivariate Cox regression analysis. Patients with missing or unknown data were excluded from the Cox model analysis. All tests were two-tailed, and statistical significance was defined as a p-value less than 0.05.

## 3 Results

**Figure 2:**
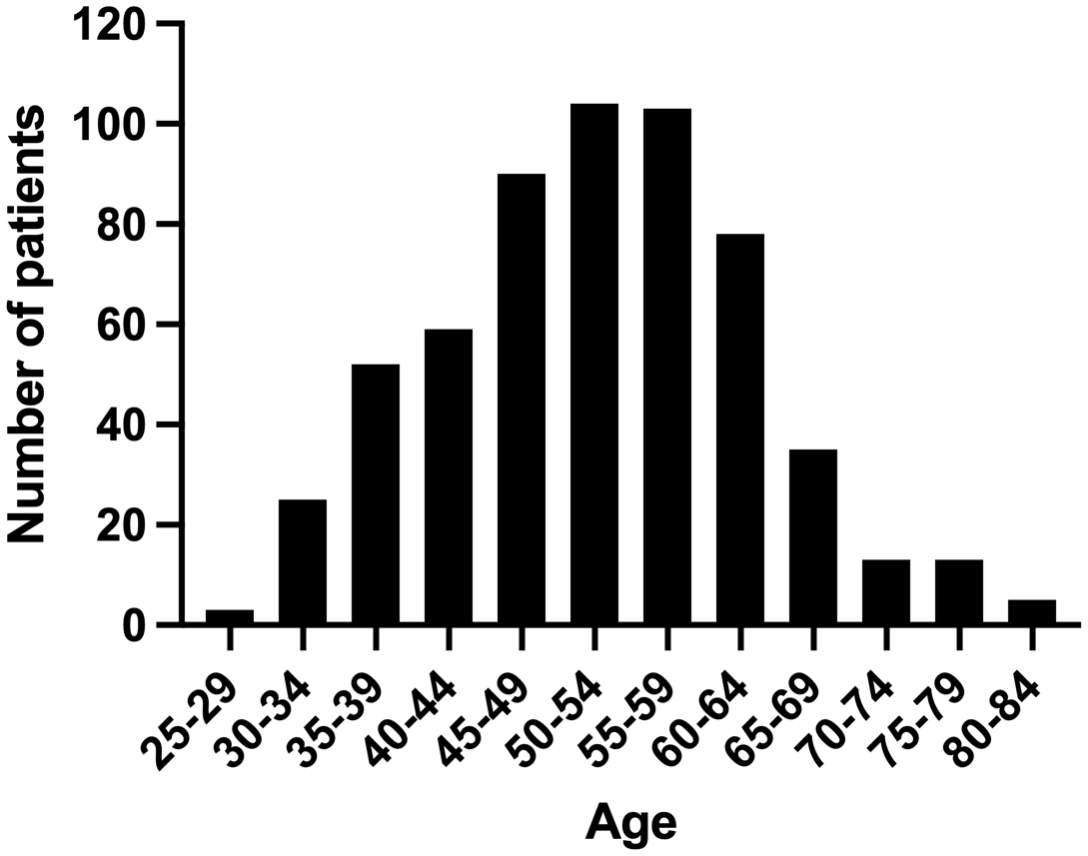
Age histograms for all patients with breast cancer treated at our institution.

During the study period, 920 BC patients underwent surgery, of which 580 patients met the inclusion criteria and comprised the analytic cohort (**Figure 1**). The median age of these 580 patients was 52 years (range: 25–81), with 80 (13.8%) categorized as AYAs and 500 (86.2%) as OAs. **Figure 2** presented the age and gender distribution histogram of the 580 patients. Using PSM, we successfully created 76 pairs of AYA and OA patients.

### 3.1 Comparisons of characteristics

The clinicopathological characteristics of AYA and OA patients before and after PSM are depicted in **Table 1**. Significant discrepancies were observed in the age at menarche between the two groups. AYA patients, as opposed to OA patients, exhibited a lower age at menarche, with the majority experiencing menarche at 15 years or younger (63.7% vs. 40.4%, p < 0.001). Similarly, substantial differences were found in the prevalence of menopausal status, with menopause being more common among OA patients compared to AYA patients (62.6% vs. 1.3%, p < 0.001). Comorbid conditions, including diabetes, hypertension, and heart disease, were more prevalent in the OA patients compared to AYA patients, with a higher prevalence of two or more comorbid conditions in OA patients as opposed to AYA patients (9.8% vs. 0%, p < 0.001). There was a difference in surgery type between the two groups, specifically, OA patients had a higher proportion of receiving mastectomy compared to AYA patients (75.2% vs. 63.7%, p = 0.031). Molecular subtype analysis revealed that among the four subtypes, the TNBC, associated with the poorest prognosis, had a higher prevalence in AYA patients, while being relatively less common in OA patients (23.8% vs. 19.4%, p = 0.046). In addition, AYA patients have a higher proportion of patients with high Ki-67 levels (61.3% vs. 47.0%, p = 0.036).

**Table 1:** Patient clinicopathologic characteristics.

	Before PSM (N = 580)			After PSM (N = 152)		
	AYA patients (N = 80)	OA patients (N = 500)	*p* value	AYA patients (N = 76)	OA patients (N = 76)	*p* value
Age, years, mean ± SD	35.7 ± 2.9	54.9 ± 8.9	< 0.001***	35.6 ± 3.0	46.1 ± 4.2	< 0.001***
Menarche, years			< 0.001***			0.737
≥ 15	29 (36.3%)	298 (59.6%)		29 (38.2%)	27 (35.5%)	
< 15	51 (63.7%)	202 (40.4%)		47 (61.8%)	49 (64.4%)	
Menopause			< 0.001***			1
Yes	1 (1.3%)	313 (62.6%)		1 (1.3%)	0 (0%)	
No	79 (98.8%)	187 (37.4%)		75 (98.7%)	76 (100%)	
Abortion	44 (55.0%)	266 (53.2%)	0.764	41 (54.0%)	44 (57.9%)	0.624
Diabetes	1 (1.3%)	50 (10.0%)	0.01*	1 (1.3%)	1 (1.3%)	1
Hypertension	0 (0%)	105 (21.0%)	< 0.001***	0 (0%)	4 (5.3%)	0.12
Heart disease	0 (0%)	42 (8.4%)	0.002**	0 (0%)	1 (1.3%)	1
Comorbid conditions ≥ 2	0 (0%)	49 (9.8%)	< 0.001***	0 (0%)	0 (0%)	NA
BMI			0.173			0.745
≤ 24	44 (55.0%)	234 (46.8%)		42 (55.3%)	40 (52.6%)	
> 24	36 (45.0%)	266 (53.2%)		34 (44.7%)	36 (47.4%)	
Tumor location			0.689			0.91
UOQ	49 (61.3%)	276 (55.2%)		46 (60.5%)	49 (64.5%)	
UIQ	13 (16.3%)	79 (15.8%)		12 (15.8%)	9 (11.8%)	
LIQ	7 (8.8%)	49 (9.8%)		7 (9.2%)	6 (7.9%)	
LOQ	6 (7.5%)	39 (7.8%)		6 (7.9%)	8 (10.5%)	
Central	5 (6.3%)	57 (11.4%)		5 (6.6%)	4 (5.3%)	
Surgery			0.031*			0.39
Mastectomy	51 (63.7%)	376 (75.2%)		48 (63.2%)	53 (69.7%)	
BCS	29 (36.3%)	124 (24.8%)		28 (36.8%)	23 (30.3%)	
Tumor size			0.715			1
≤ 2cm	44 (55.0%)	257 (51.4%)		42 (55.3%)	42 (55.3%)	
2–5cm	32 (40.0%)	223 (44.6%)		30 (39.5%)	31 (40.8%)	
> 5cm	4 (5.0%)	20 (4.0%)		4 (5.3%)	3 (4.0%)	
T stage			0.401			1
1	43 (53.8%)	256 (51.2%)		41 (54.0%)	42 (55.3%)	
2	31 (38.8%)	220 (44.0%)		29 (38.2%)	29 (38.2%)	
3	4 (5.0%)	19 (3.8%)		4 (5.3%)	3 (4.0%)	
4	2 (2.5%)	5 (1.0%)		2 (2.6%)	2 (2.6%)	
N stage			0.734			0.39
0	40 (50.0%)	275 (55.0%)		38 (50.0%)	44 (57.9%)	
1	23 (28.7%)	143 (28.6%)		22 (29.0%)	22 (29.0%)	
2	11 (13.8%)	52 (10.4%)		10 (13.2%)	4 (5.3%)	
3	6 (7.5%)	30 (6.0%)		6 (7.9%)	6 (7.9%)	
TNM stage			0.607			0.472
I	26 (32.5%)	176 (35.2%)		24 (31.6%)	27 (35.5%)	
II	35 (43.8%)	229 (45.8%)		34 (44.7%)	37 (48.7%)	
III	19 (23.8%)	95 (19.0%)		18 (23.7%)	12 (15.8%)	
Lymphatic invasion	40 (50.0%)	225 (45.0%)	0.405	38 (50.0%)	32 (42.1%)	0.329
Cancer embolus	24 (30.0%)	151 (30.2%)	0.971	23 (30.3%)	20 (26.3%)	0.589
Histological type			0.346			0.775
Ductal	56 (70.0%)	394 (78.8%)		53 (69.7%)	52 (68.4%)	
Lobular	12 (15.0%)	48 (9.6%)		12 (15.8%)	12 (15.8%)	
Mixed	5 (6.3%)	23 (4.6%)		5 (6.6%)	3 (4.0%)	
Other	7 (8.8%)	35 (7.0%)		6 (7.9%)	9 (11.8%)	
Histological grade			0.438			0.957
Grade I	7 (8.8%)	66 (13.2%)		7 (9.2%)	8 (10.5%)	
Grade II	45 (56.3%)	284 (56.8%)		44 (57.9%)	44 (57.9%)	
Grade III	28 (35.0%)	150 (30.0%)		25 (32.9%)	24 (31.6%)	
Molecular subtype			0.046*			0.161
Luminal A	8 (10.0%)	106 (21.2%)		8 (10.5%)	16 (21.1%)	
Luminal B	41 (51.2%)	199 (39.8%)		41 (54.0%)	29 (38.2%)	
Her2+	12 (15.0%)	98 (19.6%)		10 (13.2%)	10 (13.2%)	
TNBC	19 (23.8%)	97 (19.4%)		17 (22.4%)	21 (27.6%)	
ER			0.865			0.618
Positive	48 (60.0%)	305 (61.0%)		48 (63.2%)	45 (59.2%)	
Negative	32 (40.0%)	195 (39.0%)		28 (36.8%)	31 (40.8%)	
PR			0.403			0.189
Positive	48 (60.0%)	275 (55.0%)		48 (63.2%)	40 (52.6%)	
Negative	32 (40.0%)	225 (45.0%)		28 (36.8%)	36 (47.4%)	
HER2			0.647			0.864
Positive	27 (33.8%)	182 (36.4%)		25 (32.9%)	26 (34.2%)	
Negative	53 (66.3%)	318 (63.6%)		51 (67.1%)	50 (65.8%)	
Ki67			0.036*			0.139
≤ 5	4 (5.0%)	58 (11.6%)		4 (5.3%)	7 (9.2%)	
5–30	27 (33.8%)	207 (41.4%)		27 (35.5%)	36 (47.4%)	
≥ 30	49 (61.3%)	235 (47.0%)		45 (59.2%)	33 (43.4%)	
Adjuvant therapy			0.973			1
None	7 (8.8%)	57 (11.4%)		7 (9.2%)	7 (9.2%)	
CT	48 (6.0%)	286 (57.2%)		46 (60.5%)	45 (59.2%)	
RT	1 (1.3%)	7 (1.4%)		1 (1.3%)	1 (1.3%)	
ET	1 (1.3%)	8 (1.6%)		1 (1.3%)	1 (1.3%)	
Combination therapy	23 (28.7%)	142 (28.4%)		21 (27.6%)	22 (29.0%)	

AYA, adolescents and young adult; OA, older adults; PSM, propensity score match; SD, standard deviation; BMI, body mass index; UOQ, upper-outer quadrant; UIQ, upper-inner quadrant; LOQ, lower-outer quadrant; LIQ, lower-inner quadrant; BCS, breast-conserving surgery; TNBC, triple negative breast cancer; ER, estrogen receptor; PR, progesterone receptor; HER2, human epidermal growth factor receptor 2; CT, chemo therapy; RT, radio therapy; ET, endocrine therapy; NA, not applicable; *p < 0.05; **p < 0.01; ***p < 0.001.

There were no statistically significant differences between the two groups of patients in terms of BMI, tumor location, tumor size, T stage, TNM stage, lymphatic invasion, cancer embolus, histological type, histological grade, ER positivity rate, PR positivity rate, HER2 positivity rate, and adjuvant therapy. After PSM, a re-evaluation was conducted on AYA patients (N = 76) and OA patients (N = 76). The clinicopathological features were successfully matched between the AYA and OA groups, with no significant differences observed.

### 3.2 Comparisons of prognostic outcomes

**Figure 3:**
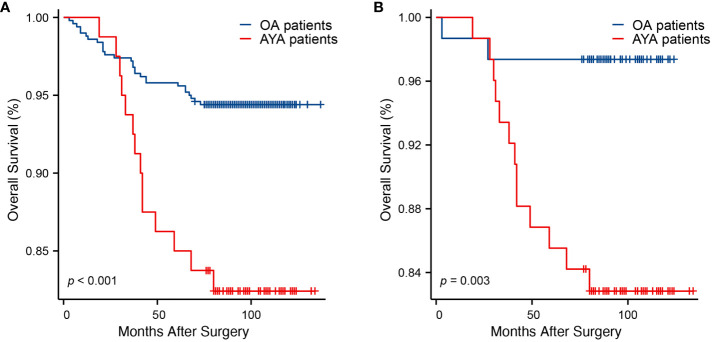
Kaplan-Meier curves of overall survival. **(A)** Before propensity score matching (p < 0.001). **(B)** After propensity score matching (p = 0.003).

Comparisons of OS between AYA and OA patients, both before and after PSM, are presented in **Table 2**. Prior to PSM, with a median follow-up of 95 months, the mortality rate in the entire AYA patient cohort was significantly higher than that of OA patients (17.5% vs. 5.6%, p < 0.001). Correspondingly, within the overall study population, the mean OS for AYA patients was notably shorter compared to OA patients (118.0 months vs. 131.3 months, p < 0.001), demonstrating a statistically significant difference (**Figure 3A**). The 1-year OS rate was similar for both AYA and OA patients (98.8% for both). However, the 3-year OS rate was slightly lower for AYA patients compared to OA patients (93.8% vs. 97.2%), and the 5-year OS rate was significantly lower for AYA patients (85.0% vs. 95.8%). Similarly, the 10-year OS rate was lower for AYA patients than for OA patients (82.4% vs. 94.4%). Following PSM, AYA patients still exhibited a significantly higher rate of mortality during follow-up compared to OA patients (17.1% vs. 2.6%, p = 0.003). The mean OS for AYA patients remained lower than that of OA patients (118.4 months vs. 121.1 months, p = 0.003) (**Figure 3B**). The 1-year and 3-year OS rates continued to be comparable between AYA and OA patients (98.7% for both at 1 year, and 93.4% vs. 97.4% at 3 years). However, the 5-year and 10-year OS rates were lower for AYA patients (85.5% at 5 years and 82.8% at 10 years) compared to OA patients (97.4% at 5 years and 10 years).

**Table 2:** Long-term OS outcomes before and after propensity score matching.

	Before PSM (N = 580)			After PSM (N = 152)		
	AYA patients (N = 500)	OA patients (N = 80)	*p* value	AYA patients (N = 76)	OA patients (N = 76)	*p* value
Death during the follow-up	14 (17.5%)	28 (5.6%)	< 0.001***	13 (17.1%)	2 (2.6%)	0.003**
Mean OS (95% CI)	118.0 (110.2 - 125.7)	131.3 (129.2 - 133.4)	< 0.001***	118.4 (110.6 - 126.3)	121.1 (117.2 - 125.1)	0.003**
1-year OS rate, %	98.8%	98.8%		98.7%	98.7%	
3-year OS rate, %	93.8%	97.2%		93.4%	97.4%	
5-year OS rate, %	85.0%	95.8%		85.5%	97.4%	
10-year OS rate, %	82.4%	94.4%		82.8%	97.4%	

AYA, adolescents and young adult; OA, older adult; OS, overall survival; CI, confidence interval; PSM, propensity score match; **p < 0.01; ***p < 0.001.

**Figure 4:**
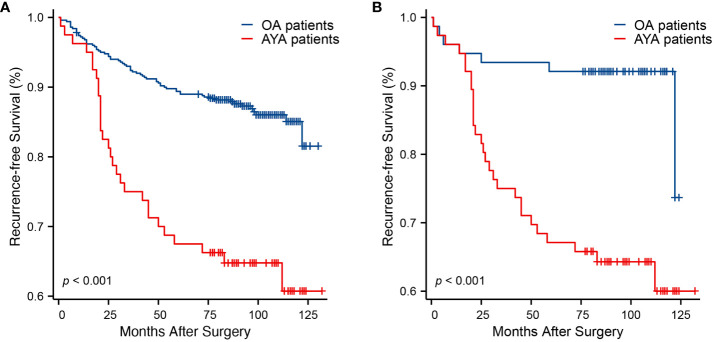
Kaplan-Meier curves of recurrence-free survival. **(A)** Before propensity score matching (p < 0.001). **(B)** After propensity score matching (p < 0.001).

Comparisons of RFS between AYA and OA patients, both before and after PSM, are presented in **Table 3**. Before PSM, over a median follow-up of 93 months, AYA patients exhibited a significantly higher recurrence rate during follow-up compared to OA patients (36.3% vs. 13.4%, p < 0.001). Similarly, before PSM, the mean RFS for AYA patients was significantly shorter than that of OA patients (95.4 months vs. 117.1 months, p < 0.001) (**Figure 4A**). The 1-year RFS rates were similar for both AYA and OA patients (96.3% vs. 97.0%). However, before PSM, the 3-year RFS rate was slightly lower for AYA patients compared to OA patients (75.0% vs. 92.4%), and the 5-year RFS rate was significantly lower for AYA patients compared to OA patients (67.5% vs. 89.4%). Similarly, the 10-year RFS rate was lower for AYA patients than for OA patients (60.7% vs. 85.1%). After PSM, AYA patients still exhibited a significantly higher recurrence rate during follow-up compared to OA patients (36.8% vs. 9.2%, p < 0.001). The mean RFS for AYA patients remained shorter than that of OA patients (95 months vs. 115.3 months, p < 0.001) (**Figure 4B**). The 1-year RFS rates remained comparable between AYA and OA patients (96.1% for both). However, after PSM, the 3-year RFS rate was still slightly lower for AYA patients compared to OA patients (75.0% vs. 93.4%), and the 5-year RFS rate remained lower for AYA patients compared to OA patients (67.1% vs. 92.1%), with the 10-year RFS rate still being lower for AYA patients compared to OA patients (60.0% vs. 92.1%).

**Table 3:** Long-term RFS outcomes before and after propensity score matching.

	Before PSM (N = 580)			After PSM (N = 152)		
	AYA patients (N = 80)	OA patients (N = 500)	*p* value	AYA patients (N = 76)	OA patients (N = 76)	*p* value
Recurrence during the follow-up	29 (36.3%)	67 (13.4%)	< 0.001***	28 (36.8%)	7 (9.2%)	< 0.001***
Mean RFS (95% CI)	95.4 (84.5 - 106.3)	117.1 (114.1 - 120.0)	< 0.001***	95.0 (83.8 - 106.2)	115.3 (108.7 - 121.8)	< 0.001***
1-year RFS rate, %	96.3%	97.0%		96.1%	96.1%	
3-year RFS rate, %	75.0%	92.4%		75.0%	93.4%	
5-year RFS rate, %	67.5%	89.4%		67.1%	92.1%	
10-year RFS rate, %	60.7%	85.1%		60.0%	92.1%	

AYA, adolescents and young adult; OA, older adult; RFS, recurrence-free survival; CI, confidence interval; PSM, propensity score match; ***p < 0.001.

### 3.3 Prognostic analyses

Conducting univariate and multivariate Cox proportional hazards regression analyses on the cohort before PSM, young age (15–39 years) emerged as an independent risk factor for OS (HR 2.659, 95% CI 1.385–5.106, p = 0.003) and RFS (HR 3.235, 95% CI 2.085–5.022, p < 0.001) in BC patients after surgery. Other independent predictors of OS included BMI and TNM stage (**Table 4**). Other independent predictors of RFS included surgery and TNM stage (**Table 5**). After PSM, young age (15–39 years) remained an independent risk factor for OS (HR 5.736, 95% CI 1.278–25.735, p = 0.023) and RFS (HR 6.009, 95% CI 2.424–14.895, p < 0.001) in BC patients after surgery. Other independent predictors of OS were menopause and T stage (**Table 6**). Additionally, for RFS, other independent predictors included menopause, surgery, and T stage (**Table 7**).

**Table 4:** Univariate and multivariate Cox regression analysis of overall survival after mastectomy for breast cancer before propensity matching.

Variables		Univariate		Multivariate	
		HR (95% CI)	*p* value	HR (95% CI)	*p* value
Age	AYA vs. OA	3.264 (1.718–6.201)	< 0.001***	2.659 (1.385–5.106)	0.003**
Menarche, years	≤ 15 vs. > 15	0.703 (0.374–1.321)	0.273		
Menopause	Yes vs. No	1.066 (0.582–1.953)	0.836		
Abortion	Yes vs. No	0.645 (0.35–1.188)	0.16		
Comorbid conditions	≥ 2 vs. < 2	1.148 (0.41–3.216)	0.793		
BMI	> 24 vs. ≤ 24	0.553 (0.297–1.031)	0.062	0.49 (0.26–0.924)	0.028*
Tumor location					
	UOQ	Reference	0.445		
	UIQ	0.878 (0.359–2.147)	0.775		
	LIQ	0.955 (0.331–2.752)	0.932		
	LOQ	1.833 (0.749–4.484)	0.184		
	Central	0.43 (0.102–1.817)	0.251		
Surgery	BCS vs. Mastectomy	0.285 (0.102–0.799)	0.017*	NA	0.159
T stage					
	1	Reference	0.003**	Reference	0.901
	2	2.81 (1.383–5.711)	0.004**	NA	0.654
	3	6.262 (2.175–18.024)	0.001**	NA	0.749
	4	4.413 (0.57–34.179)	0.155	NA	0.627
N stage					
	0	Reference	< 0.001***	Reference	0.797
	1	3.64 (1.452–9.123)	0.006**	NA	0.511
	2	10.304 (4.11–25.831)	< 0.001***	NA	0.708
	3	12.676 (4.72–34.046)	< 0.001***	NA	0.627
TNM stage					
	I	Reference	< 0.001***	Reference	< 0.001***
	II	5.863 (1.341–25.637)	0.019*	5.798 (1.326–25.357)	0.02*
	III	24.791 (5.871–104.679)	< 0.001***	25.588 (6.043–108.341)	< 0.001***
Cancer embolus	Yes vs. No	3.599 (1.944–6.666)	< 0.001***	NA	0.744
Histological type					
	Ductal	Reference	0.902		
	Lobular	1.187 (0.463–3.041)	0.721		
	Mixed	0.979 (0.235–4.079)	0.977		
	Other	0.63 (0.151–2.624)	0.525		
Histological grade					
	Grade I	Reference	0.385		
	Grade II	1.73 (0.52–5.763)	0.372		
	Grade III	2.27 (0.661–7.79)	0.193		
Molecular subtype					
	Luminal A	Reference	0.372		
	Luminal B	2.295 (0.781–6.745)	0.131		
	Her2+	2.131 (0.642–7.078)	0.217		
	TNBC	2.778 (0.885–8.725)	0.08		
ER	Negative vs. Positive	1.305 (0.711–2.397)	0.39		
PR	Negative vs. Positive	1.407 (0.768–2.578)	0.269		
HER2	Negative vs. Positive	0.732 (0.397–1.348)	0.316		
ki67					
	≤ 5	Reference	0.123		
	5–30	1.066 (0.301–3.776)	0.921		
	≥ 30	2.032 (0.616–6.698)	0.244		
Adjuvant therapy					
	None	Reference	0.536		
	CT	0.594 (0.237–1.487)	0.266		
	RT	1.304 (0.157–10.834)	0.806		
	ET	0 (0–4.199E+254)	0.97		
	Combination therapy	1.016 (0.397–2.595)	0.974		

AYA, adolescents and young adult; OA, older adults; HR, hazards ratio; CI, confidence interval; BMI, body mass index; UOQ, upper-outer quadrant; UIQ, upper-inner quadrant; LOQ, lower-outer quadrant; LIQ, lower-inner quadrant; BCS, breast-conserving surgery; TNBC, triple negative breast cancer; ER, estrogen receptor; PR, progesterone receptor; HER2, human epidermal growth factor receptor 2; CT, chemo therapy; RT, radio therapy; ET, endocrine therapy; NA, not applicable; *p < 0.05; **p < 0.01; ***p < 0.001.

**Table 5:** Univariate and multivariate Cox regression analysis of recurrence-free survival after mastectomy for breast cancer before propensity matching.

Variables		Univariate		Multivariate	
		HR (95% CI)	*p* value	HR (95% CI)	*p* value
Age	AYA vs. OA	3.069 (1.984–4.749)	< 0.001***	3.235 (2.085–5.022)	< 0.001***
Menarche, years	≤ 15 vs. > 15	0.874 (0.581–1.313)	0.516		
Menopause	Yes vs. No	0.993 (0.665–1.484)	0.973		
Abortion	Yes vs. No	1.05 (0.702–1.568)	0.813		
Comorbid conditions	≥ 2 vs. < 2	0.861 (0.399–1.858)	0.703		
BMI	> 24 vs. ≤ 24	0.923 (0.619–1.378)	0.696		
Tumor location					
	UOQ	Reference	0.868		
	UIQ	1.238 (0.733–2.091)	0.426		
	LIQ	0.971 (0.479–1.968)	0.934		
	LOQ	0.952 (0.433–2.094)	0.902		
	Central	0.799 (0.38–1.681)	0.554		
Surgery	BCS vs. Mastectomy	0.411 (0.229–0.738)	0.003**	0.499 (0.272–0.914)	0.024*
T stage					
	1	Reference	< 0.001***	Reference	0.092
	2	1.641 (1.059–2.542)	0.027*	NA	0.479
	3	3.827 (1.894–7.734)	< 0.001***	NA	0.355
	4	7.112 (2.525–20.032)	< 0.001***	NA	0.023*
N stage					
	0	Reference	< 0.001***	Reference	0.63
	1	1.62 (0.982–2.671)	0.059	NA	0.88
	2	3.038 (1.733–5.328)	< 0.001***	NA	0.236
	3	4.713 (2.566–8.657)	< 0.001***	NA	0.209
TNM stage					
	I	Reference	< 0.001***	Reference	< 0.001***
	II	1.818 (1.028–3.214)	0.038*	1.673 (0.943–2.965)	0.078
	III	4.382 (2.466–7.785)	< 0.001***	3.714 (2.064–6.683)	< 0.001***
Cancer embolus	Yes vs. No	1.97 (1.317–2.946)	0.001**	NA	0.851
Histological type					
	Ductal	Reference	0.346		
	Lobular	0.712 (0.344–1.473)	0.359		
	Mixed	0.569 (0.18–1.801)	0.337		
	Other	0.495 (0.181–1.35)	0.17		
Histological grade					
	Grade I	Reference	0.015*	Reference	0.078
	Grade II	3.167 (1.147–8.746)	0.026*	NA	0.623
	Grade III	4.313 (1.539–12.086)	0.005**	NA	0.097
Molecular subtype					
	Luminal A	Reference	0.141		
	Luminal B	2.026 (1.04–3.947)	0.038*		
	Her2+	2.041 (0.977–4.262)	0.058		
	TNBC	2.261 (1.101–4.642)	0.026*		
ER	Negative vs. Positive	1.276 (0.853–1.909)	0.235		
PR	Negative vs. Positive	1.16 (0.777–1.732)	0.467		
HER2	Negative vs. Positive	0.771 (0.514–1.157)	0.209		
ki67					
	≤ 5	Reference	0.026*	Reference	0.11
	5–30	1.298 (0.571–2.953)	0.534	NA	0.099
	≥ 30	2.149 (0.977–4.729)	0.057	NA	0.036*
Adjuvant therapy					
	None	Reference	0.456		
	CT	2.257 (0.905–5.632)	0.081		
	RT	3.277 (0.636–16.901)	0.156		
	ET	0 (0–1.898E+154)	0.956		
	Combination therapy	2.378 (0.924–6.116)	0.072		

AYA, adolescents and young adult; OA, older adults; HR, hazards ratio; CI, confidence interval; BMI, body mass index; UOQ, upper-outer quadrant; UIQ, upper-inner quadrant; LOQ, lower-outer quadrant; LIQ, lower-inner quadrant; BCS, breast-conserving surgery; TNBC, triple negative breast cancer; ER, estrogen receptor; PR, progesterone receptor; HER2, human epidermal growth factor receptor 2; CT, chemo therapy; RT, radio therapy; ET, endocrine therapy; NA, not applicable; *p < 0.05; **p < 0.01; ***p < 0.001.

**Table 6:** Univariate and multivariate Cox regression analysis of overall survival after mastectomy for breast cancer after propensity matching.

Variables		Univariate		Multivariate	
		HR (95% CI)	*p* value	HR (95% CI)	*p* value
Age	AYA vs. OA	6.818 (1.538–30.218)	0.012*	5.736 (1.278–25.735)	0.023*
Menarche, years	≤ 15 vs. > 15	0.362 (0.129–1.018)	0.054	NA	0.051
Menopause	Yes vs. No	0.007 (0–0.106)	< 0.001***	0.007 (0–0.123)	0.001**
Abortion	Yes vs. No	0.685 (0.249–1.89)	0.466		
BMI	> 24 vs. ≤ 24	0.411 (0.131–1.29)	0.128		
Tumor location					
	UOQ	Reference	0.98		
	UIQ	0.813 (0.18–3.669)	0.788		
	LIQ	0.627 (0.081–4.853)	0.654		
	LOQ	0.605 (0.078–4.689)	0.631		
	Central	0	0.985		
Surgery	BCS vs. Mastectomy	0.49 (0.138–1.736)	0.269		
T stage					
	1	Reference	0.002**	Reference	0.003**
	2	1.186 (0.362–3.886)	0.778	1.429 (0.414–4.936)	0.573
	3	10.133 (2.842–36.126)	< 0.001***	10.678 (2.837–40.195)	< 0.001***
	4	0	0.986	0	0.985
N stage					
	0	Reference	0.025*	Reference	0.364
	1	1.867 (0.467–7.465)	0.377	NA	0.790
	2	6.593 (1.647–26.396)	0.008**	NA	0.584
	3	5.779 (1.293–25.83)	0.022*	NA	0.142
TNM stage					
	I	Reference	0.007**	Reference	0.054
	II	4.377 (0.527–36.355)	0.172	NA	0.844
	III	15.507 (1.938–124.057)	0.01*	NA	0.086
Cancer embolus	Yes vs. No	2.323 (0.842–6.407)	0.103		
Histological type					
	Ductal	Reference	0.953		
	Lobular	0.817 (0.181–3.684)	0.792		
	Mixed	1.228 (0.159–9.515)	0.844		
	Other	0.611 (0.079–4.731)	0.637		
Histological grade					
	Grade I	Reference	0.146		
	Grade II	0.415 (0.08–2.137)	0.293		
	Grade III	1.255 (0.267–5.913)	0.774		
Molecular subtype					
	Luminal A	Reference	0.594		
	Luminal B	2.082 (0.251–17.295)	0.497		
	Her2+	3.773 (0.392–36.278)	0.25		
	TNBC	3.28 (0.383–28.082)	0.278		
ER	Negative vs. Positive	1.857 (0.673–5.122)	0.232		
PR	Negative vs. Positive	1.607 (0.583–4.431)	0.36		
HER2	Negative vs. Positive	0.549 (0.199–1.515)	0.247		
ki67					
	≤ 5	Reference	0.091	Reference	0.326
	5–30	0.353 (0.032–3.896)	0.396	NA	0.189
	≥ 30	1.836 (0.239–14.125)	0.559	NA	0.139
Adjuvant therapy					
	None	Reference	0.549		
	CT	1.285 (0.161–10.274)	0.813		
	RT	7.685 (0.48–123.093)	0.15		
	ET	0	0.988		
	Combination therapy	1.655 (0.193–14.166)	0.646		

AYA, adolescents and young adult; OA, older adults; HR, hazards ratio; CI, confidence interval; BMI, body mass index; UOQ, upper-outer quadrant; UIQ, upper-inner quadrant; LOQ, lower-outer quadrant; LIQ, lower-inner quadrant; BCS, breast-conserving surgery; TNBC, triple negative breast cancer; ER, estrogen receptor; PR, progesterone receptor; HER2, human epidermal growth factor receptor 2; CT, chemo therapy; RT, radio therapy; ET, endocrine therapy; NA, not applicable; *p < 0.05; **p < 0.01; ***p < 0.001.

**Table 7:** Univariate and multivariate Cox regression analysis of recurrence-free survival after mastectomy for breast cancer after propensity matching.

Variables		Univariate		Multivariate	
		HR (95% CI)	*p* value	HR (95% CI)	*p* value
Age	AYA vs. OA	4.477 (1.953–10.261)	< 0.001***	6.009 (2.424–14.895)	< 0.001***
Menarche, years	≤ 15 vs. > 15	0.671 (0.345–1.306)	0.24		
Menopause	Yes vs. No	0.047 (0.006–0.385)	0.004**	0.029 (0.003–0.273)	0.002**
Abortion	Yes vs. No	1.05 (0.538–2.051)	0.886		
BMI	> 24 vs. ≤ 24	1.506 (0.773–2.933)	0.229		
Tumor location					
	UOQ	Reference	0.55		
	UIQ	0.907 (0.346–2.379)	0.843		
	LIQ	0.271 (0.037–2.007)	0.201		
	LOQ	0.506 (0.119–2.14)	0.354		
	Central	1.494 (0.448–4.98)	0.513		
Surgery	BCS vs. Mastectomy	0.465 (0.203–1.065)	0.07	0.361 (0.145–0.897)	0.028*
T stage					
	1	Reference	0.001**	Reference	< 0.001***
	2	0.966 (0.448–2.084)	0.93	0.909 (0.414–1.992)	0.811
	3	3.736 (1.364–10.236)	0.01*	2.93 (1.036–8.286)	0.043*
	4	7.005 (2.026–24.221)	0.002**	20.495 (5.133–81.838)	< 0.001***
N stage					
	0	Reference	0.044*	Reference	0.379
	1	1.606 (0.719–3.588)	0.248	NA	0.881
	2	3.05 (1.158–8.03)	0.024*	NA	0.855
	3	3.388 (1.206–9.518)	0.021*	NA	0.102
TNM stage					
	I	Reference	0.002**	Reference	0.288
	II	1.787 (0.692–4.612)	0.23	NA	0.567
	III	4.813 (1.847–12.546)	0.001**	NA	0.48
Cancer embolus	Yes vs. No	1.584 (0.797–3.149)	0.189		
Histological type					
	Ductal	Reference	0.808		
	Lobular	1.01 (0.412–2.475)	0.982		
	Mixed	0.956 (0.226–4.042)	0.952		
	Other	0.488 (0.115–2.062)	0.329		
Histological grade					
	Grade I	Reference	0.12		
	Grade II	1.436 (0.331–6.236)	0.629		
	Grade III	2.735 (0.629–11.901)	0.18		
Molecular subtype					
	Luminal A	Reference	0.932		
	Luminal B	1.106 (0.403–3.033)	0.844		
	Her2+	0.88 (0.236–3.279)	0.849		
	TNBC	1.267 (0.43–3.727)	0.668		
ER	Negative vs. Positive	0.999 (0.507–1.966)	0.997		
PR	Negative vs. Positive	0.862 (0.438–1.696)	0.666		
HER2	Negative vs. Positive	0.995 (0.493–2.006)	0.988		
ki67					
	≤ 5	Reference	0.491		
	5–30	0.522 (0.168–1.621)	0.261		
	≥ 30	0.703 (0.238–2.076)	0.523		
Adjuvant therapy					
	None	Reference	0.81		
	CT	1.704 (0.398–7.294)	0.472		
	RT	3.958 (0.358–43.709)	0.262		
	ET	0 (0–6.73E+289)	0.976		
	Combination therapy	2.067 (0.46–9.276)	0.343		

AYA, adolescents and young adult; OA, older adults; HR, hazards ratio; CI, confidence interval; BMI, body mass index; UOQ, upper-outer quadrant; UIQ, upper-inner quadrant; LOQ, lower-outer quadrant; LIQ, lower-inner quadrant; BCS, breast-conserving surgery; TNBC, triple negative breast cancer; ER, estrogen receptor; PR, progesterone receptor; HER2, human epidermal growth factor receptor 2; CT, chemo therapy; RT, radio therapy; ET, endocrine therapy; NA, not applicable; *p < 0.05; **p < 0.01; ***p < 0.001.

## 4 Discussion

BC is relatively uncommon in adults aged 40 and younger. However, in recent years, the incidence of BC in younger women, defined as AYAs, has been rising. Approximately one in every 300 women will receive a BC diagnosis before the age of 40 (19). It’s noteworthy that young women are not the primary focus of screening programs. Organized screening of young, healthy women has been widely acknowledged as inefficient and, in some cases, even potentially harmful by most experts (20, 21). As a result, BC in younger women tends to be diagnosed at more advanced stages, resulting in poorer clinical outcomes and more treatment complications compared to older patients (22). Currently, there are limited clinical studies on AYA patients with BC. This study compared the clinical pathological characteristics and prognosis outcomes between 80 AYA patients and 500 OA patients after BC surgery, mainly exploring the influence of age of onset on BC prognosis.

Menarche and menopause mark the commencement and conclusion of ovarian activity associated with reproduction, respectively, and they exert an influence on BC risk. The earlier women experience menarche, the higher their subsequent risk of BC becomes. Moreover, the impact of being 1 year younger at menarche on BC risk is notably more significant than that of being 1 year older at menopause (23). Additional research has indicated that the risk of developing early-onset BC at the age of 45 or younger is higher in women who experienced menarche at the age of 12, compared to women whose first menstruation occurred at 15 or older (24). The risk of early-onset BC increases with every year younger at the age of menarche, and the premenopausal status is also linked to an elevated risk of early-onset BC (25). Additionally, the mean age at menarche was lower in women under age 40 (26). Our findings are consistent with existing literature, indicating that AYA women with BC are typically premenopausal. Although their age at menopause cannot be determined at present and compared with OA patients, AYA patients have a younger age at menarche. Furthermore, compared to OA patients, AYA patients have fewer comorbid conditions, possibly due to their younger age. Surgical intervention results in enduring physiological modifications to the patient’s body. The decision to pursue breast-conserving therapy as a local treatment for young women diagnosed with early-stage BC is a topic of controversy. Notably, breast conserving surgery (BCS) has demonstrated survival advantages that are at least comparable to those of mastectomy, with even more favorable outcomes observed in patients aged 36 to 40 years (27). Eleven studies assessed the psychological impact of breast cancer surgery on adolescent and AYA patients. Several studies have consistently indicated that mastectomy is linked to poorer quality of life aspects, including body image, sexual well-being, and heightened anxiety, in contrast to patients who have undergone BCS (28–30). Many patients choose to undergo mastectomy because of concerns about the possibility of cancer cells being left behind and a desire to avoid future screening imaging (31). BCS was more commonly performed in the younger age group of Chinese women (26). Our study revealed that more than half of AYA patients opted for mastectomy, although the proportion of those choosing BCS was comparatively higher than that of OA patients, which aligns with findings from prior research.

Significantly, the typical presentation of advanced stages at the time of diagnosis, along with more aggressive pathological features, a higher incidence of triple-negative and HER2-overexpressing tumors, increased rates of recurrence at any clinical stage, and inferior OS rates compared to older women, are the primary factors contributing to the ‘aggressive’ nature of BC in young women (11, 32). Our cohort demonstrated that the AYA group exhibited more aggressive cancer subtypes compared to the OA group, with 23.8% being triple negative, 61.3% showing high Ki-67 levels, and a higher proportion having stage 3+ disease, thus confirming the aforementioned points. Some retrospective studies have shown a worse 5-year survival in young women with BC compared with older. This subgroup of patients has different risk factors, tumor biology, poorer prognosis (33, 34). In summary, among the differing clinical and pathological characteristics in the two patient groups, factors such as age at menarche, menstrual status, molecular subtypes, Ki-67 levels, comorbidities, and others were influential in BC risk or prognosis. Our study revealed that, in comparison to the OA group, the AYA group demonstrates a higher incidence of adverse clinical outcomes, which aligns with prior research findings. Our additional analyses of prognosis before PSM suggested that these factors likely contribute to OS and RFS differences between AYA and OA groups. After balancing potential prognostic factors via PSM, AYA group continued to show lower OS and RFS rates compared to OA group. Furthermore, results from a multivariate Cox regression analysis provided additional evidence that young age (15–39 years) serves as an independent risk factor for both OS and RFS in BC patients after surgery.

Since BC in younger women is associated with poorer survival rates, early detection has the potential to enhance survival, minimize invasive treatments, and improve overall quality of life, thus reducing the burden of disease and the costs of treatment (35). Consequently, it is essential to follow diagnostic and treatment guidelines specifically addressed to young women. Despite providing valuable insights, this study has several limitations. As a single-center retrospective analysis, it may introduce selection bias and institution-specific results, necessitating further multicenter studies to ensure the reliability of the findings. Although PSM was used to reduce confounding factors, the relatively small sample size of AYA patients may limit statistical power. Additionally, since all patients in this study received treatment in China, larger-scale studies are needed to ensure the generalizability of the findings to a broader patient population. Studies have shown that genetic mutations can significantly impact treatment decisions and prognosis for BC patients, particularly in younger patients. Compared to older women, early-onset breast cancers are more commonly associated with BRCA gene mutations or other genetic susceptibilities. Young-BRCA mutation carriers, compared to non-carriers, exhibit poorer prognosis (36). Understanding the mutation status of BRCA and other related susceptibility genes is of crucial importance for treatment planning (including bilateral prophylactic mastectomy) (37, 38). However, due to the current low prevalence of genetic testing among BC patients, we lack information on the genetic testing results of the patients in this study. Consequently, this study does not reflect the impact of genetic factors. Further comprehensive evaluations of treatment strategies and prognostic factors of AYA patients are needed to provide age-specific care recommendations. Overall, this study emphasized the significance of young age as an independent risk factor for OS and RFS in BC patients, even after PSM. Age-specific care and management strategies should be considered to enhance the clinical outcomes of AYA patients with BC. Further research is essential to enhance our understanding of the unique challenges and needs of this patient population.

## 5 Conclusions

Compared to OA patients, AYA patients with BC tend to have a younger age at menarche, a lower prevalence of menopausal status, a decreased frequency of mastectomy, a higher incidence of TNBC, elevated Ki-67 levels, and a reduced occurrence of comorbid conditions. In terms of prognosis outcomes, within the study cohort, both before and after PSM, AYAs exhibited higher mortality rates and poorer long-term survival compared to OAs. In the PSM-adjusted cohort, the mean OS and RFS for AYAs were significantly shortened. Young age (15–39 years) emerged as an independent risk factor for OS and RFS in BC patients following surgery.

## Data availability statement

The raw data supporting the conclusions of this article will be made available by the authors, without undue reservation.

## Ethics statement

The studies involving humans were approved by Institutional Review Board of the Qingdao Central Hospital. The studies were conducted in accordance with the local legislation and institutional requirements. The participants provided their written informed consent to participate in this study.

## Author contributions

XL: Formal analysis, Writing – original draft. ZM: Data curation, Writing – original draft. HC: Software, Writing – original draft. WN: Methodology, Writing – original draft. GS: Visualization, Writing – original draft. KZ: Writing – review & editing. XZ: Project administration, Supervision, Writing – review & editing.
