# Corrigendum: Long-term oncologic outcomes following breast cancer surgery in adolescents and young adults: a single-center retrospective analysis

**DOI:** 10.3389/fonc.2024.1481345

**Published:** 2024-09-19

**Authors:** Xin Liu, Zengyan Ma, Hongwu Chu, Weihong Nie, Guoxin Sun, Kaihua Zhao, Xiao Zou

**Affiliations:** ^1^ Qingdao Medical College, Qingdao University, Qingdao, China; ^2^ Department of Pathology, Qingdao Central Hospital, Qingdao, China; ^3^ Guangdong Provincial Key Laboratory of Digestive Cancer Research, The Seventh Affiliated Hospital of Sun Yat-sen University, Shenzhen, China; ^4^ Department of Breast Surgery, Qingdao Central Hospital, Qingdao, China; ^5^ Department of Breast Surgery, Xiangdong Hospital Affiliated to Hunan Normal University, Liling, China

**Keywords:** breast cancer, age, young, clinicopathological characteristics, prognosis

In the published article, there was an error in **Table 2** as published. The term “Median OS” were incorrectly used. The correct term should be “Mean OS,” as the statistical analysis was based on mean values. The corrected **Table 2** and its caption appear below.

**Table 2:** Long-term OS outcomes before and after propensity score matching.

	Before PSM (N = 580)			After PSM (N = 152)		
	AYA patients (N = 500)	OA patients (N = 80)	*p* value	AYA patients (N = 76)	OA patients (N = 76)	*p* value
Death during the follow-up	14 (17.5%)	28 (5.6%)	< 0.001***	13 (17.1%)	2 (2.6%)	0.003**
Mean OS (95% CI)	118.0 (110.2 - 125.7)	131.3 (129.2 - 133.4)	< 0.001***	118.4 (110.6 - 126.3)	121.1 (117.2 - 125.1)	0.003**
1-year OS rate, %	98.8%	98.8%		98.7%	98.7%	
3-year OS rate, %	93.8%	97.2%		93.4%	97.4%	
5-year OS rate, %	85.0%	95.8%		85.5%	97.4%	
10-year OS rate, %	82.4%	94.4%		82.8%	97.4%	

AYA, adolescents and young adult; OA, older adult; OS, overall survival; CI, confidence interval; PSM, propensity score match; **p < 0.01; ***p < 0.001.

In the published article, there was an error in **Table 3** as published. The term “Median RFS” were incorrectly used. The correct term should be “Mean RFS,” as the statistical analysis was based on mean values. The corrected **Table 3** and its caption appear below.

**Table 3:** Long-term RFS outcomes before and after propensity score matching.

	Before PSM (N = 580)			After PSM (N = 152)		
	AYA patients (N = 80)	OA patients (N = 500)	*p* value	AYA patients (N = 76)	OA patients (N = 76)	*p* value
Recurrence during the follow-up	29 (36.3%)	67 (13.4%)	< 0.001***	28 (36.8%)	7 (9.2%)	< 0.001***
Mean RFS (95% CI)	95.4 (84.5 - 106.3)	117.1 (114.1 - 120.0)	< 0.001***	95.0 (83.8 - 106.2)	115.3 (108.7 - 121.8)	< 0.001***
1-year RFS rate, %	96.3%	97.0%		96.1%	96.1%	
3-year RFS rate, %	75.0%	92.4%		75.0%	93.4%	
5-year RFS rate, %	67.5%	89.4%		67.1%	92.1%	
10-year RFS rate, %	60.7%	85.1%		60.0%	92.1%	

AYA, adolescents and young adult; OA, older adult; RFS, recurrence-free survival; CI, confidence interval; PSM, propensity score match; ***p < 0.001.

In the published article, there was an error. The terms “median OS” and “median RFS” were incorrectly used in the original article. The correct terms should be “mean OS” and “mean RFS,” as the statistical analysis was based on mean values.

A correction has been made to **Abstract**, *Results*, line 8. This section previously stated:

“median OS”

The corrected term appears below:

“mean OS”

A correction has been made to *Comparisons of prognostic outcomes*, the first paragraph, lines 6 and 17. This section previously stated:

“median OS”

The corrected term appears below:

“mean OS”

A correction has been made to *Comparisons of prognostic outcomes*, second paragraph, lines 6 and 16. This section previously stated:

“median RFS”

The corrected term appears below:

“mean RFS”

A correction has been made to **Conclusions**, the first paragraph, line 8. This section previously stated:

“median OS”

The corrected term appears below:

“mean OS”

The authors apologize for these errors and state that this does not change the scientific conclusions of the article in any way. The original article has been updated.

